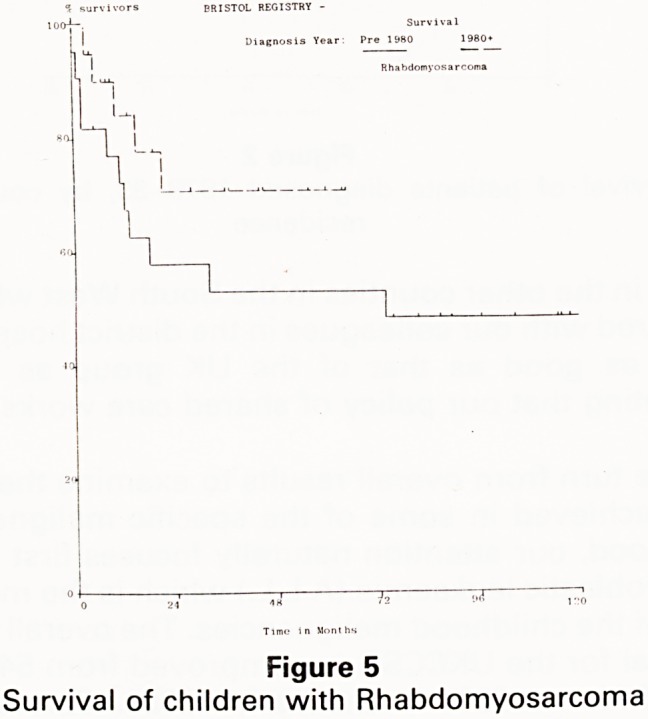# Current Prognosis for Childhood Cancer in the South West Region

**Published:** 1988

**Authors:** M. G. Mott

**Affiliations:** Bristol Children's Hospital


### Current Prognosis in Childhood Malignancy

**M. G. Mott**
Bristol Children's Hospital

The outlook for children with cancer has altered remarkably in the last twenty years, making one of the most dramatic changes in medical history, perhaps second only to the change in mortality from bacterial infectious diseases which occurred with the advent of antibiotics a generation earlier.

The incidence of childhood cancers has remained stable according to the most reliable data now available, and is about one hundred cases per million children per year throughout North America and Western Europe. This means that about one child in every six hundred develops cancer, a total of twelve hundred new cases each year in the U.K., or one child with cancer in twenty years' practice for the average general practitioner. The change in prognosis is well illustrated by observing the national death rate for childhood cancer, which fell from 7.3 per hundred thousand in 1960 to 4.7 per hundred thousand in 1979, representing an effective doubling of the survival rate in that short period of time (1).

**Table 1**

**Annual death rate per 100,000 (G.B.)**
**Age 0–14 years**

| *Year* | *Rate* | *Survivors* |
|---|---|---|
| 1961 | 7.28 | 27.2% |
| 1965 | 7.05 | 29.5% |
| 1970 | 6.49 | 35.1% |
| 1975 | 5.31 | 46.9% |
| 1979 | 4.64 | 53.6% |

Increase in survivors 1961–1979=50% OPCS, 1982

Many factors have contributed to this substantial improvement in survival rates. The importance of some of these is emphasised by comparing the outcome of different groups of children treated within the United Kingdom in recent years. The improvement in the 1970's is largely attributable to the development of a multi-disciplinary team approach in a number of the major paediatric teaching hospitals, and their increasing collaboraton with one another and with colleagues abroad in both national and international therapeutic trials and studies. This development led to the formation in 1977, of the United Kingdom Children's Cancer Study Group (UKCCSG). From its formation the group has registered the majority of children with malignancy treated in the U.K., rising to about 70% by 1986. This means some 30% of children with cancer are still treated outside the group, a factor which is important when we review the comparative survival rates for the two groups.

Bristol is one of the largest of the centres which make up the UKCCSG and our experience is in many respects typical. The actuarial five year survival for our patients treated in the 1970s was 53% and this has risen to over 60% in the 1980s, which is a most encouraging overall result (Fig 1). We have already published data indicating that the actuarial 5 year survival rate for children diagnosed in 1979–83 and living in Avon County, who received essentially all of their treatment at the Children's Hospital was 67% (2): the survival rate for the children

**Figure 1**
Survival of all patients treated, by time of diagnosis

**Figure 2**
Survival of patients diagnosed 1979–83, by county of residence living in the other counties in the South West whose care is shared with our colleagues in the district hospitals was 53%, as good as that of the UK group as a whole, indicating that our policy of shared care works well (Fig 2).

If we turn from overall results to examine the survival rates achieved in some of the specific malignancies of childhood, our attention naturally focuses first on acute lymphoblastic leukaemia (A.L.L.) which is the most common of the childhood malignancies. The overall five year survival for the UKCCSG has improved from 54% in the late 1970s to 65% in the early 1980s. Corresponding figures for Bristol show a five year survival of 60% for all patients treated throughout the 1970's, increasing in the 1980s to 76%, a truly remarkable change from the very small percentage of survivors in the late 1960s and early 1970s, before the advent of prospective randomised clinical trials under the auspices of the Medical Research Council (Fig 3).

Results for the childhood non-Hodgkins lymphomas (NHL) are equally gratifying (3,4). Bristol patients achieved a five year survival of 63% in the 1970s and

**Figure 3**
Survival of children with A.L.L. by time of diagnosis

**Figure 4**
Survival of children with N.H.L.

**Figure 5**
Survival of children with Rhabdomyosarcoma

72% in the 1980s (Fig 4). Corresponding figures for the UKCCSG show a five year survival of 48% in the late 1970s, rising to 60% in the early 1980s, with a projected 5 year survival which will probably achieve 75% for the patients treated since 1983. These results contrast starkly with a survival rate of only 35% achieved by other teaching hospitals for patients in the early 1980s in the UK (5).

The treatment of the sarcomas of bone and soft tissues is another area where substantial headway has been achieved. The survival rate for Bristol patients with Rha domyosarcoma in the 1970s for example is 53%, rising 71% for those diagnosed in the 1980s (Fig 5). The figures mirror closely those achieved by the UKCCSG a whole, and are substantially better than those achieve for children treated in other centres (6).

### DISCUSSION

A wealth of data now exists to substantiate the marke improvements in survival achieved for children wi malignant disease, an improvement that is clearly re lated to the development of multi-disciplinary team ca in the major teaching hospitals, and to the advent large national and international collaborative venture We have passed beyond the stage where a mere majori of children become long term survivors and curren results suggest that at least two-thirds of the childre diagnosed can be expected to achieve long term surviv if they are given appropriate treatment. It is sobering recall however that a substantial minority of these chil ren are still not offered optimal treatment, and the prognosis has been documented to be much worse (6

Regular critical analysis of results shows that the tren for improvement continues without slackening, and th enables us to feel confident that we are still on the rig track. There are a number of selected groups of childre in whom an 80–90% survival rate can now be attaine and for these groups substantial progress has bee made in simplifying their treatment, thus reducing th long term sequelae while maintaining their exceller prospects of cure. There can no longer be any excuse fo occasional dabbling in this field, for the consequences o doing so are clearly documented: not just an increase mortality, but also severe and unnecessary morbidity fo the survivors.

The political and managerial implications are als clear. It is unacceptable that, while some regional healt authorities have accepted their responsibility to provid an efficient, centralised service for their patients, other continue to rely on charities to support most of th required services while forcing the district health autho ities into arguments about who should pay for wha proportion of each patients' care. There cannot be man situations where the consequences of failing to exert th necessary managerial impetus are so clearly defined fo all to see (7).

### REFERENCES

1. DRAPER, G. J., BIRCH, J. M., BITHELL, J. F. et al. (198 Childhood Cancer in Britain. Studies on Medical and Popula tion Subjects No 37., O.P.C.S. pp29–39.

2. MOTT, M. G., OAKHILL, A., EDEN, O. B. (1985) Shared Ca for Children with Cancer. British Paediatric Association C15 pp104.

3. MOTT, M. G., EDEN, O. B., PALMER, M. K. (1985) Adjuvan low dose radiation in childhood non-Hodgkins Lymphom *Brit.J.Cancer* Vol 50 pp463–469.

4. MOTT, M. G., CHESSELLS, J. M., WILLOUGHBY, M. N. L (1984) et al. Adjuvant low dose radiation in childhood T ce leukaemia/lymphoma *BritJ.Cancer* Vol 50 pp457–402.

5. MOTT, M. G., EDEN, O.B., STILLER, C. A. (1986). Improve Survival in Childhood non-Hodgkins Lymphoma—Nationa impact of the UKCCSG Trial. British Paediatric Associatio p21 pp47.

6. STILLER, C. A. (1988). Increased centralisation of treatme and improvements in survival rates for cancer in Brita 1977–84. *ArchDisChildhood.* Vol 63 pp23–30.

7. Cancer Services for Children: report to the DHSS from th United Kingdom Childrens Cancer Study Group (UKCCSG). 1987.

## Figures and Tables

**Figure 1 f1:**
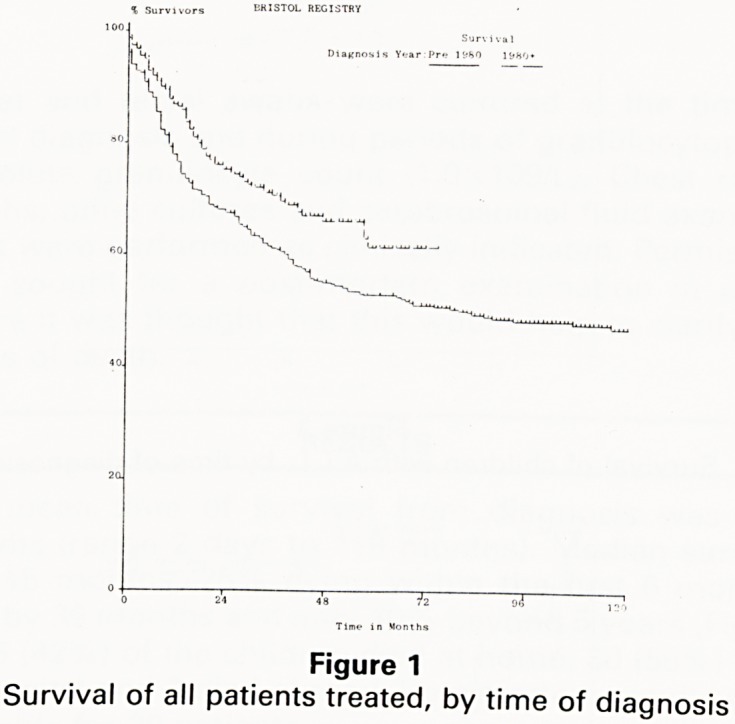


**Figure 2 f2:**
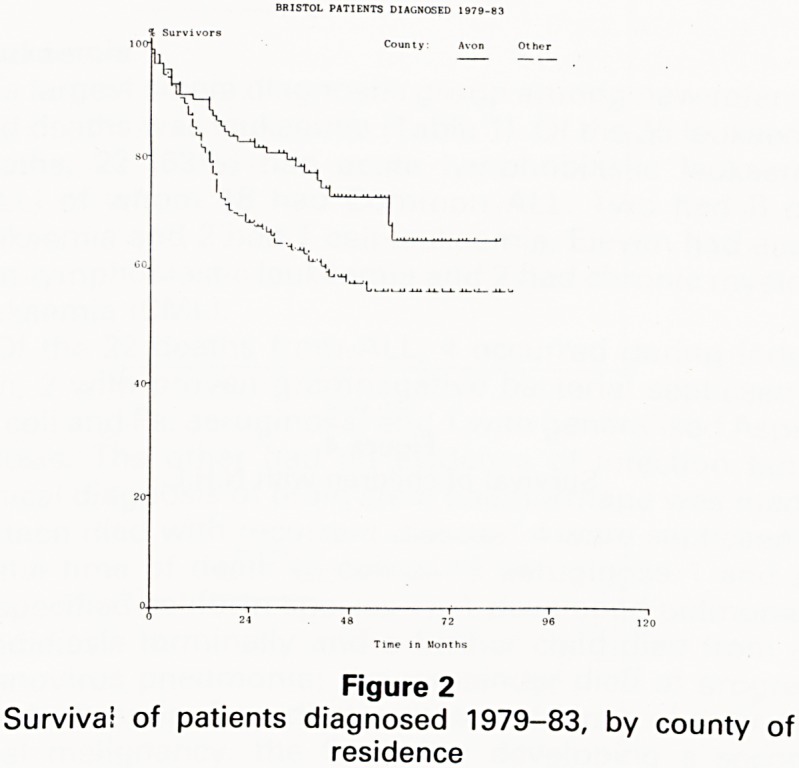


**Figure 3 f3:**
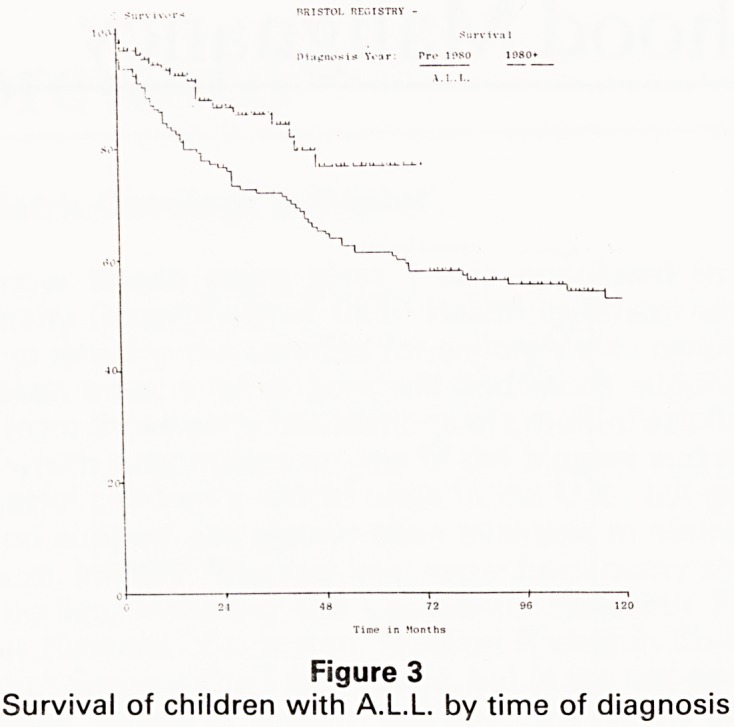


**Figure 4 f4:**
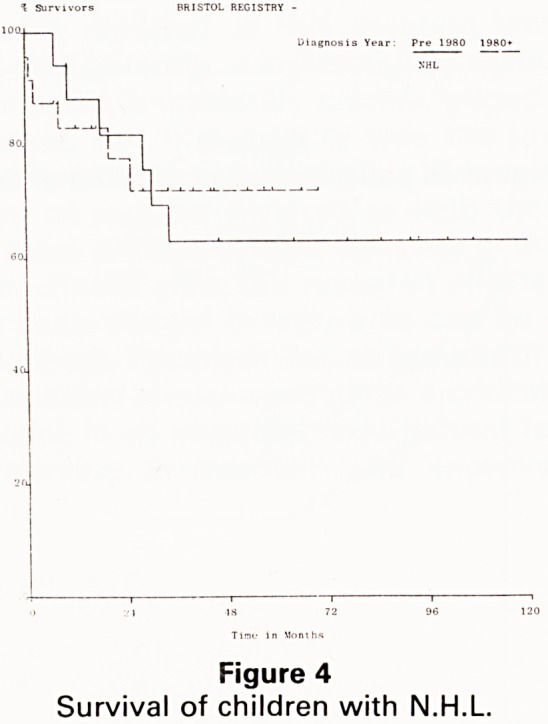


**Figure 5 f5:**